# Ergot-Poisoning

**Published:** 1880-11-20

**Authors:** John M. Keating

**Affiliations:** Consulting Accoucheur to Philadelphia Hospital; Lecturer on Diseases of Children in University of Pennsylvania, etc.


### ERGOT-POISONING.

BY JOHN M. KEATING, M.D.,

Consulting Accoucheur to Philadelphia Hospital; Lecturer on Diseases of Children in University of Pennsylvania, etc.

The following case presents certain features of interest, and I do not remember to have read of one like it in any of our own or foreign journals.

I was engaged to attend Mrs. D—— in her confinement to come off the first week in the current month, as it eventually did.

The family had moved to the city from a country town some years ago, and Mrs. D. was placed under my care for uterine disease. She had some inflammatory trouble following a previous labor. After a short course of the usual treatment she entirely recovered, and soon after became a second time pregnant.

At the third month she over-fatigued herself by some house-cleaning duties, and a miscarriage resulted. I was absent from the city at the time, and upon my return at the end of the summer, found my patient relapsed into her former state, with side-ache, purulent uterine discharge, subinvolution and its accompaniments. Once more she regained her normal condition, and again became pregnant. As the uterus enlarged there were evidences of "binding down," probably from some old adhesions about the left ovarian region. For some weeks previous to confinement she was unable to leave the house, for the abdomen was very much enlarged. There was great flatulence, and the patient suffered continually from left sciatica. The child was a large one, but the pelvis was capacious.

Fearing some difficulty from uterine inertia, I explained her case to a medical friend, and urged her to send at once for him, should the messenger find me absent from my office. As is usual in these cases, the child came at an inopportune time, but my friend arrived early enough to save the patient considerable pain by the application of the short forceps of Simpson. The head had well descended, and was resting at the outlet, but the uterus was unable to contract sufficiently to produce expulsion.

There were placental adhesions of great firmness, and in consequence more than the ordinary amount of hemorrhage.

At last the uterus was well emptied, the binder applied, and ʒ ij. of the fluid extract of ergot administered—this by the doctor himself.

The patient was left comfortable, with instructions to the nurse to send for the doctor at once in case of hemorrhage, and while the messenger was absent to give the patient ʒ ss. of the ergot every half-hour till the doctor's return. By a misunderstanding the ʒ ss. of ergot was administered every half-hour from the time the doctor left. I reached the house a few moments after the messenger had been sent in search of me, and found my patient presenting an appearance that was indeed alarming. The face was of a blueish tint, and she seemed in great pain. The pupils were dilated, the pulse was quick, very weak, and occasionally irregular; there was dyspnœa, nausea (no vomiting), buzzing in the ears, and at times a tendency to syncope. The skin was cool and clammy. I was informed that another baby was expected.

Upon inquiry, I learnt that in all she had taken about ℥ ss. of the fluid extract of ergot (and this was afterward corroborated by the medical attendant from the amounts left in the bottle which he himself had brought to the house). I loosened the binder, lowered her head, gave her some whiskey, and stimulated the circulation by rubbing, and in the space of half an hour the severity of the symptoms had gradually passed, and patient was left to sleep off a dose of morphia and potass. bromide that was administered.

One of the most interesting features in the case was the powerful uterine contractions. This alone was so marked as to have silenced in my own mind any doubts as to the efficiency of ergot, had I ever been a skeptic on the subject.—*Medical Record.*

